# P02.193. Yoga of awareness: a randomized trial in fibromyalgia: post intervention and 3 month follow up results

**DOI:** 10.1186/1472-6882-12-S1-P249

**Published:** 2012-06-12

**Authors:** C Wright, J Carson, K Carson, R Bennett, S Mist, K Jones

**Affiliations:** 1 Oregon Health & Science University, Portland, Oregon, USA

## Purpose

Comprehensive fibromyalgia (FM) treatment requires medications, exercise and improvement of coping skills. However, existing exercise protocols exert inadequate analgesic effects and suffer from poor adherence. The central hypothesis of the study is that yoga practiced with concurrent substantive mindfulness will reduce pain-related fear, increase pain acceptance and pressure pain thresholds, resulting in long-term adherence.

## Methods

Fifty-three women with FM were randomized to an 8 week RCT of 2 hours weekly supervised group yoga + mindfulness or wait listed control. Yoga + mindfulness consisted of gentle poses, meditation, breathing exercises, yoga-based coping instructions, and group discussions drawing strongly on the Kripalu school of yoga.

## Results

Immediately post-intervention women assigned to the yoga + mindfulness program compared to wait listed controls showed significantly greater improvements on standardized measures of FM symptoms and functioning (Revised Fibromyalgia Impact Questionnaire, FIQR), including pain, pain pressure thresholds, fatigue, mood, pain catastrophizing, acceptance, and other coping strategies. Post-treatment results in the wait-list group largely mirrored results seen at post-treatment in the immediate treatment group, with the FIQR Total Score improving 31.9% across the two groups. Follow-up results showed that patients sustained most of their post-treatment gains, with the FIQR total score remaining 21.9% improved at 3 months. Multilevel random effects models demonstrated that those who practiced more had greater improvements. Home practice was 31 minutes daily vs 40 minutes daily in the wait listed vs immediately treated groups, respectively.

## Conclusion

This novel pilot RCT indicated that yoga + mindfulness may be a safe, effective and durable intervention for women with FM.

